# Variability in SCAT6 Dual Action Tandem Gait Performance Across Athletic Disciplines: Implications for Concussion Assessment

**DOI:** 10.51894/001c.143947

**Published:** 2025-09-30

**Authors:** Jacob Kostecke, Nathan Fitton

**Affiliations:** 1 College of Osteopathic Medicine Michigan State University

## 27

### INTRODUCTION

The Sports Concussion Assessment Tool 6 (SCAT6) Dual Action Tandem Gait test evaluates cognitive-motor integration post-concussion. This study investigated performance variability across athletic disciplines, hypothesizing that athletes in sports requiring balance, agility, and cognitive coordination (e.g., baseball, softball) would outperform those in strength/endurance-focused disciplines (e.g., basketball, rowing).

### HYPOTHESIS

We aimed to assess how an athlete’s discipline profile and cognitive-motor demands influence their test performance, positing that sport-specific profiles and skills would correlate with improved outcomes.

### METHODS

Eighty-one MSU athletes (balance/agility: baseball, cheer, softball; strength/endurance: basketball, rowing, swimming, track & field, wrestling) completed three heel-to-toe walking trials while serially subtracting 7s from three starting numbers (88, 90, 98). Performance metrics included completion time and counting errors. Data on sports discipline, previous history of lower extremity injury or surgery, and biological sex were analyzed.

### RESULTS

Balance/agility-sport athletes reduced times (55.84s→50.35s→46.61s, n=31) but had stable errors (1.00→0.97→1.03). Strength/endurance-sport athletes improved times (65.68s→59.28s→55.44s, n=50) and reduced errors (1.44 →0.98→0.92). Athletes improved completion times across trials. Male athletes reduced times (54.52s→48.10s→42.81s, n=31) but showed fluctuating errors (0.87→0.77→0.94). Female athletes improved both times (66.50s→60.68s→57.80s, n=50) and errors (1.52→1.10→0.98). No significant performance differences were observed for athletes with prior lower extremity injuries or surgeries.

### DISCUSSION

The findings partially support our hypothesis: while balance/agility-sport athletes achieved faster times, their error patterns contrasted with strength/endurance athletes, who prioritized accuracy as trials progressed. Biological sex differences emerged, with males favoring speed (despite error fluctuations) and females demonstrating progressive accuracy. The lack of injury-related performance deficits suggests cognitive-motor tasks may not be influenced by prior physical trauma. However, sport-specific cognitive demands (e.g., baseball’s repetitive decision-making vs. rowing’s endurance focus) may explain divergent error trends. The third trial’s starting number (98, a multiple of 7) likely reduced cognitive load, contributing to time improvements across all groups.

### CONCLUSION

These results underscore the need for concussion assessments tailored to sport-specific cognitive profiles and biological sex-based strategies. Standardizing cognitive tasks (e.g., fixed starting numbers) and controlling environmental distractions could enhance diagnostic consistency. Future studies should expand cohorts to validate trends and explore longitudinal training effects.

